# Matrix Metalloproteinases in Glioma: Drivers of Invasion and Therapeutic Targets

**DOI:** 10.3390/biotech14020028

**Published:** 2025-04-15

**Authors:** Ella E. Aitchison, Alexandra M. Dimesa, Alireza Shoari

**Affiliations:** 1 Department of Cancer Biology, Mayo Clinic, Jacksonville, FL 32224, USA; ella.aitchison@student.manchester.ac.uk (E.E.A.); dimesa.alexandra@mayo.edu (A.M.D.); 2 School of Biological Sciences, Faculty of Biology Medicine and Health, University of Manchester, Manchester M13 9PT, UK; 3 Department of Biology, University of North Florida, Jacksonville, FL 32224, USA

**Keywords:** matrix metalloproteinases (MMPs), gliomas, glioblastoma, tumor invasion, therapeutic targeting

## Abstract

Matrix metalloproteinases (MMPs) are a family of zinc-dependent proteolytic enzymes that are crucial for the remodeling of the extracellular matrix, a process that is often co-opted by cancers, including brain tumors, to facilitate growth, invasion, and metastasis. In gliomas, MMPs contribute to a complex interplay involving tumor proliferation, angiogenesis, and immune modulation, thereby influencing tumor progression and patient prognosis. This review provides a comprehensive analysis of the roles of various MMPs in different types of gliomas, from highly malignant gliomas to metastatic lesions. Emphasis is placed on how the dysregulation of MMPs impacts tumor behavior, the association between specific MMPs and the tumor grade, and their potential as biomarkers for diagnosis and prognosis. Additionally, the current therapeutic approaches targeting MMP activity are discussed, exploring both their challenges and future potential. By synthesizing recent findings, this paper aims to clarify the broad significance of MMPs in gliomas and propose avenues for translational research that could enhance treatment strategies and clinical outcomes.

## 1. Introduction

Brain cancer encompasses a wide spectrum of malignant tumors originating in the central nervous system (CNS), including primary brain tumors like gliomas, meningiomas, and medulloblastomas, as well as secondary tumors from metastatic spread [1,2]. Despite advances in neurosurgical techniques, radiotherapy, and chemotherapy, the prognosis for malignant brain tumors remains poor, especially for high-grade gliomas, such as glioblastoma multiforme (GBM) [3]. The complex and aggressive nature of these tumors stems, in part, from their ability to invade surrounding tissue, resist apoptosis, induce angiogenesis, and evade immune surveillance [4]. These properties are, to a significant degree, facilitated by MMPs, which are a family of zinc-dependent endopeptidases involved in extracellular matrix (ECM) remodeling [5]. MMPs are a group of enzymes that are capable of degrading various components of the ECM, which provides structural and biochemical support to surrounding cells [6]. Originally recognized for their roles in physiological processes like wound healing, tissue remodeling, and development, MMPs have since been implicated in pathological conditions, including cancer, where they play a role in promoting tumor cell invasion, the spread of metastasis, and angiogenesis [7]. The 23 human MMPs identified to date are classified based on their structural domains and substrate specificity, with subgroups including collagenases, gelatinases, stromelysins, and membrane-type MMPs [8].

In the context of cancer, MMPs facilitate tumor progression by degrading ECM barriers, thus enabling cancer cells to invade adjacent tissue and spread to distant sites [9]. Beyond ECM degradation, MMPs also modulate the tumor microenvironment through the release of growth factors, cytokines, and chemokines from the ECM, promoting angiogenesis and enhancing immune evasion [10]. Dysregulation in MMP expression has been observed in various malignancies, and specific MMPs have been linked to poor clinical outcomes, underscoring their potential as both biomarkers and therapeutic targets [11]. Gliomas exhibit unique physiological and microenvironmental characteristics that make them particularly challenging to treat, and MMPs play vital roles in this challenge by enabling critical processes in tumor progression, including breakdown of the blood–brain barrier, which facilitates cancer cell migration and immune cell infiltration into the CNS [12]. Notably, MMP-2 [13] and MMP-9 [14] have been associated with glioma invasiveness, while other MMPs, such as MMP-1 [15] and MMP-7, are linked to metastatic gliomas [16]. Understanding how these MMPs operate within the unique CNS environment is essential to developing targeted interventions for brain tumors.

Due to their roles in cancer invasion and metastasis, MMPs are increasingly recognized for their potential as prognostic biomarkers and therapeutic targets [17]. Elevated MMP levels have been correlated with higher tumor grades, an increased risk of recurrence, and reduced survival in patients with gliomas [18]. By identifying and quantifying specific MMPs within tumor tissue, cerebrospinal fluid, and serum, clinicians may be able to improve early diagnosis, predict patient outcomes, and tailor therapies more effectively. However, the clinical application of MMP-targeted therapies has been limited by challenges related to specificity and adverse effects, making further research essential to harness MMPs’ full potential in glioma treatment [19].

A systematic literature search was conducted to ensure the comprehensive coverage of relevant studies. The search utilized the PubMed, Scopus, and Web of Science databases, focusing on peer-reviewed articles. The search strategy included combinations of keywords such as “matrix metalloproteinases”, “glioma”, “glioblastoma”, “tumor invasion”, “angiogenesis”, and “therapeutic targeting”. Studies were included if they provided insights into the roles of MMPs in glioma biology, clinical or preclinical data on MMPs as biomarkers or therapeutic targets, or mechanistic details regarding MMP regulation in gliomas. The exclusion criteria involved non-English articles, studies unrelated to MMPs or gliomas, and non-peer-reviewed sources such as conference abstracts or editorials. The selected literature was synthesized through thematic analysis to identify consensus findings, contradictory evidence, and translational relevance.

This review aims to provide a comprehensive overview of MMPs’ roles in gliomas, synthesizing findings from molecular studies, preclinical experiments, and clinical research. We examine MMPs’ structure and function, their mechanistic contributions to glioma progression, and their potential as biomarkers and therapeutic targets. Additionally, we highlight recent advances and ongoing challenges in MMP-targeted therapies, proposing directions for future research to improve glioma treatment outcomes.

## 2. Structure and Function of MMPs

**Figure 1:**
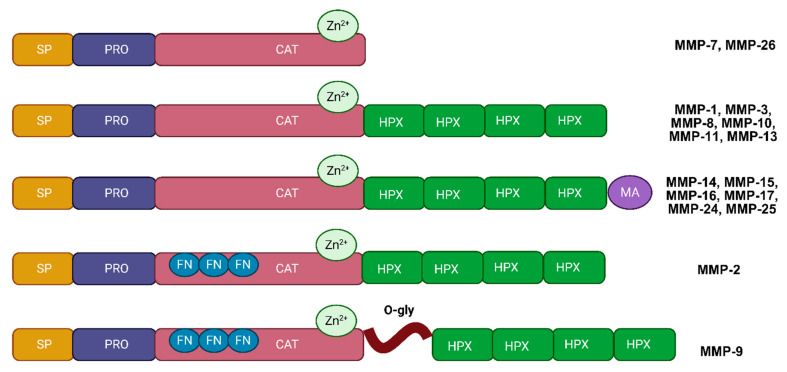
MMPs’ multidomain structure. MMPs are categorized into four groups based on their domain architecture. They share a common core structure consisting of a signal peptide (SP), a pro-domain (Pro), a catalytic domain (CAT) containing a zinc (Zn)-binding site, and a hemopexin domain (HPX). However, specific variations exist among subtypes. Gelatinases (MMP-2 and MMP-9) have three fibronectin repeats (FN) within their catalytic domain. Uniquely, MMP-9 contains a central O-glycosylated domain (O-gly), which is a flexible 64-amino-acid linker between the catalytic domain and the hemopexin domain. Additionally, some membrane-type MMPs are tethered to the membrane via a membrane anchor (MA) (illustration created with BioRender.com) (https://app.biorender.com, accessed on 6 April 2025).

MMPs are a family of zinc-dependent enzymes that are responsible for degrading ECM proteins, playing an essential role in both normal physiological processes and pathological conditions, including cancer [20] (Table 1). In gliomas, MMPs contribute significantly to tumor invasion, ECM remodeling, and alterations within the tumor microenvironment [21]. Structurally, MMPs share several key domains that enable them to perform these diverse functions (Figure 1). These domains include the pro-domain, catalytic domain, hemopexin-like domain, and, in some cases, a transmembrane domain [6]. The pro-domain keeps MMPs in an inactive form by blocking the active site, and this region must be cleaved for the enzyme to become active [22]. The catalytic domain, containing a zinc-binding motif, is the core site responsible for breaking down ECM components [23]. The hemopexin-like domain provides substrate specificity and mediates interactions with tissue inhibitors of metalloproteinases (TIMPs), which regulate MMP activity [24]. For membrane-type MMPs (MT-MMPs), an additional transmembrane domain anchors the enzyme to the cell surface, allowing localized ECM degradation, which is particularly relevant to cancer cell invasion and migration [25].

MMPs can be classified into several subgroups based on their structural domains and substrate preferences, including collagenases, gelatinases, stromelysins, membrane-type MMPs, and other specialized MMPs [26]. Collagenases, such as MMP-1, MMP-8, and MMP-13, primarily target fibrillar collagens, the most abundant proteins in the ECM [27]. Gelatinases, including MMP-2 and MMP-9, degrade denatured collagen (gelatin) and type IV collagen, which are critical components of basement membranes [28]. The degradation of basement membranes by gelatinases facilitates cancer cell invasion and metastasis, making these MMPs particularly relevant to gliomas [29]. Stromelysins, such as MMP-3 and MMP-10, degrade various ECM molecules, including proteoglycans and fibronectin, and, although their roles in gliomas are less studied, they are known to influence tissue remodeling and the availability of growth factors [30]. Membrane-type MMPs, such as MMP-14 (MT1-MMP), are anchored to the cell membrane, contributing to pericellular matrix degradation, cell migration, and angiogenesis, all of which are pivotal in invasive cancers [31]. Other MMPs, including MMP-7 and MMP-12, serve more specialized functions. For example, MMP-7 has been linked to cancer cell migration and immune modulation, while MMP-12 is involved in inflammation and has roles in certain cancers [32].

MMPs are synthesized as inactive proenzymes, or zymogens, and require activation to function. In healthy tissue, MMP activity is tightly regulated; however, in cancer, including brain tumors, this balance often becomes dysregulated, resulting in excessive MMP activity that promotes tumor progression [33]. There are several mechanisms by which MMPs become activated, including the proteolytic cleavage of the pro-domain by other proteases, such as plasmin, or even other MMPs. This is particularly relevant in the tumor microenvironment, where protease activity is elevated [34]. Chemical activation by reactive oxygen species (ROS) and other small molecules can also activate certain MMPs, like MMP-2 and MMP-9, linking oxidative stress to increased MMP activity [35]. Membrane-type MMPs, like MMP-14, can activate pro-MMPs at the cell surface, facilitating pericellular matrix degradation, which is essential for tumor invasion and metastasis [25].

In addition to degrading the ECM, MMPs perform a variety of roles that significantly influence tumor biology. They enable tumor invasion and migration by breaking down ECM components and basement membranes, which allows cancer cells to infiltrate surrounding tissue and, in advanced cases, spread to distant sites [17]. MMPs also promote angiogenesis, an essential process for tumor growth, by releasing pro-angiogenic factors like vascular endothelial growth factor (VEGF) from the ECM. By stimulating the formation of new blood vessels, MMPs ensure an adequate supply of nutrients and oxygen to the tumor, supporting its expansion [36]. Additionally, MMPs play a role in immune modulation by processing cytokines and chemokines, which can alter immune cell recruitment and activity, contributing to an immunosuppressive tumor microenvironment that facilitates immune evasion by the cancer cells [10]. Finally, MMPs actively reshape the tumor microenvironment by altering the ECM’s stiffness and structure, affecting the behavior of both tumor cells and surrounding stromal and immune cells. In cancer, these changes create a microenvironment that supports tumor progression and complicates therapeutic interventions [37].

The structure, activation, classification, and diverse functions of MMPs underscore their significance in the progression of gliomas. By facilitating key processes such as invasion, angiogenesis, immune evasion, and microenvironmental remodeling, MMPs serve as central players in glioma biology, making them promising targets for therapeutic interventions.

**Table 1:** Overview of matrix metalloproteinases (MMPs), including their family members, domain structures, cell sources, and primary biological functions.

MMP Family	Cell Source	Tissue Specificity	Function	Clinical Associations	Ref.
MMP-1 (Collagenase-1)	Fibroblasts, keratinocytes	Tumor cells, stromal fibroblasts	Degrades interstitial collagen (types I, II, III), ECM remodeling	Correlates with invasiveness, metastasis, poor prognosis	[38]
MMP-2 (Gelatinase A)	Endothelial cells, fibroblasts	Tumor cells, endothelial cells	Degrades gelatin, collagen IV, laminin; involved in angiogenesis and metastasis	High tumor grade, increased recurrence, reduced survival	[13]
MMP-3 (Stromelysin-1)	Fibroblasts, macrophages	Tumor stroma, macrophages	Degrades ECM components (e.g., proteoglycans, laminin); activates other MMPs	Potential role in tumor invasion and ECM remodeling	[39]
MMP-7 (Matrilysin)	Epithelial cells	Tumor epithelial cells	Degrades ECM components (e.g., elastin, fibronectin); involved in tissue repair and inflammation	Increased proliferation, metastasis; poor clinical outcomes	[40]
MMP-8 (Collagenase-2)	Neutrophils	Tumor-associated neutrophils	Degrades collagen types I, II, and III; involved in inflammatory processes	Associated with inflammation-related tumor progression	[41]
MMP-9 (Gelatinase B)	Neutrophils, macrophages	Tumor cells, endothelial cells, immune cells	Degrades gelatin, collagen IV, elastin; plays a role in cancer metastasis and inflammation	Poor prognosis, therapy resistance, correlation with high grades	[14]
MMP-10 (Stromelysin-2)	Fibroblasts, macrophages	Tumor-associated stromal cells, immune cells	Degrades proteoglycans, gelatin; involved in wound healing and tissue remodeling	Linked to tumor invasiveness and tissue remodeling	[42]
MMP-11 (Stromelysin-3)	Fibroblasts, tumor cells	Stromal fibroblasts, tumor-associated fibroblasts	Processes ECM components; implicated in cancer cell invasion	Poor prognosis, associated with aggressive tumor behavior	[43]
MMP-12 (Macrophage Elastase)	Macrophages	Tumor-infiltrating macrophages	Degrades elastin; involved in tissue remodeling and inflammation	Associated with inflammation-driven tumor progression	[44]
MMP-13 (Collagenase-3)	Chondrocytes, fibroblasts	Tumor-associated stromal cells, tumor cells	Degrades type II collagen; important in cartilage remodeling and osteoarthritis	Linked to tumor invasiveness, aggressiveness, poor outcomes	[45]
MMP-14 (MT1-MMP)	Fibroblasts, tumor cells	Tumor cell membrane-localized	Degrades collagen types I, II, III; activates MMP-2; involved in cell migration	Inverse correlation with survival, enhanced invasiveness	[46]
MMP-15 (MT2-MMP)	Tumor cells	Tumor cell membranes	Involved in ECM remodeling and cancer metastasis	Associated as anti-apoptotic factor in cancer cells	[47]
MMP-16 (MT3-MMP)	Tumor cells, fibroblasts	Tumor-associated cells, cell surface	Activates MMP-2; involved in ECM remodeling and angiogenesis	Linked to increased angiogenesis and invasive phenotype	[48]
MMP-17 (MT4-MMP)	Macrophages, tumor cells	Tumor cell surfaces, immune cells	ECM remodeling; implicated in cancer progression	Correlated with tumor aggressiveness	[49]
MMP-19	Keratinocytes, fibroblasts	Tumor-associated fibroblasts, stromal cells	Degrades ECM components like collagen IV, laminin; involved in tissue repair	Associated with higher tumor grades and ECM alterations	[50]
MMP-20 (Enamelysin)	Odontoblasts	Dental tissue	Specific to enamel development; degrades amelogenin	-	[51]
MMP-23	Tumor cells	Tumor tissue	Implicated in cancer progression; potential regulatory role in ECM	Potential role in tumor progression	[52]
MMP-24 (MT5-MMP)	Neurons, tumor cells	Neural tissue, tumor cell membranes	Involved in neural development and ECM remodeling	Elevated in brain tumors, potential prognostic marker	[53]
MMP-25 (MT6-MMP)	Leukocytes	Immune cells	ECM remodeling; role in inflammation	Possible role in tumor-associated inflammation	[49]
MMP-26 (Endometase)	Endometrial cells, tumor cells	Tumor epithelial cells	Degrades ECM components; implicated in reproductive tissue remodeling and cancer	Linked to aggressive cancer behaviors	[54]
MMP-28 (Epilysin)	Keratinocytes, macrophages	Epithelial and immune cells	ECM remodeling; involved in wound healing	Emerging marker for tumor progression	[55]

Abbreviations: ECM, extracellular matrix; MMPs, matrix metalloproteinases.

## 3. Roles of MMPs in Glioma Progression

### 3.1. Tumor Invasion

**Figure 2:**
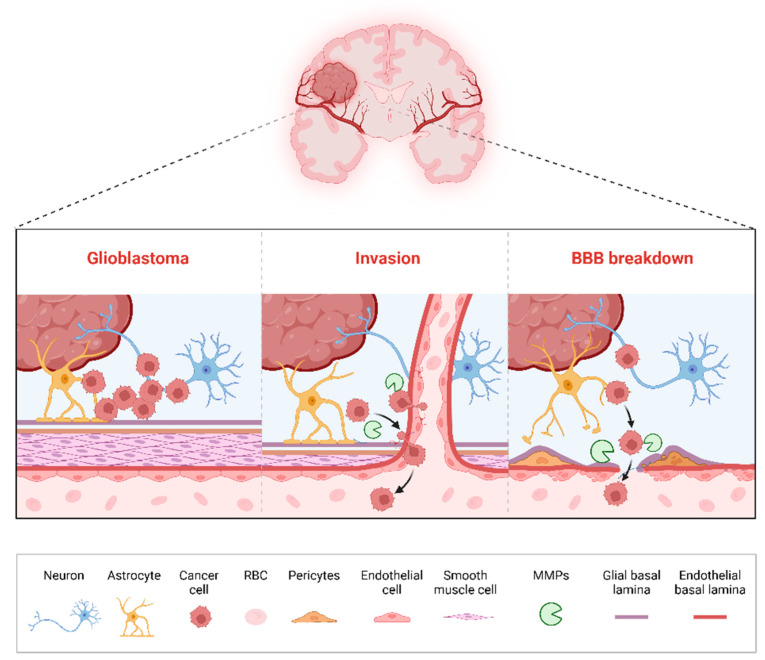
Illustration of MMP function in GBM progression, highlighting tumor growth, invasive behavior, and BBB breakdown. The sequence depicts GBM cells infiltrating the surrounding brain tissue, aided by MMPs, leading to the structural disruption of the BBB and facilitating further tumor spread. The black arrows indicate the direction of cancer cell migration from the primary tumor site toward and across the compromised BBB. Key cellular and extracellular components involved in this process are labeled. BBB, blood–brain barrier; RBC, red blood cell (illustration created with BioRender.com) (https://app.biorender.com, accessed on 6 April 2025).

MMPs play a pivotal role in the progression of gliomas, including the most aggressive and prevalent form of primary gliomas, as well as metastatic brain tumors (Figure 2) [12]. Through a variety of mechanisms, MMPs drive tumor cell invasion, angiogenesis, immune modulation, and overall alterations in the tumor microenvironment. In gliomas, where cellular infiltration into adjacent brain tissue is a hallmark of malignancy, MMPs facilitate the breakdown of ECM components and the breach of the blood–brain barrier (BBB), both of which are critical for tumor spread and resistance to treatment [56]. Understanding these roles is crucial in developing therapies that can address the aggressive nature of brain tumors and improve patient outcomes.

A key aspect of MMPs’ function in gliomas is their ability to degrade ECM proteins, allowing tumor cells to invade nearby tissue. This invasion process is particularly important in the CNS, where gliomas rarely form clear boundaries, infiltrating diffusely into the surrounding brain tissue [57]. This invasive characteristic of brain tumors makes surgical removal challenging, as cancer cells spread extensively into regions that control vital functions [58]. MMPs such as MMP-2 and MMP-9, both classified as gelatinases, are especially active in degrading type IV collagen, a primary structural component of basement membranes [59]. Their activity facilitates the breakdown of the basement membrane at the tumor–brain interface, enabling cancer cells to penetrate neighboring tissue. High levels of MMP-2 and MMP-9 have been consistently correlated with increased invasiveness in high-grade gliomas, underscoring their importance in tumor spread [60,61]. Additionally, MMP-1, a collagenase that targets fibrillar collagens in the ECM, has been implicated in facilitating cell migration and invasion in GBM [38]. The study by Ricci et al. examined the activity of MMP-2 and MMP-9 in the sera of brain tumor patients (gliomas, meningiomas, and metastases) compared to healthy controls using zymography and immunohistochemistry. While MMP-2 activity showed no significant differences, MMP-9 activity (both monomeric and multimeric forms) was significantly higher in tumor samples (*p* < 0.001). Immunohistochemistry revealed strong MMP-9 expression in the neoplastic vessels of high-grade gliomas and cytoplasmic reactivity in meningiomas, with increased expression in atypical meningiomas (*p* = 0.036). The MMP-9 levels correlated with Ki-67, suggesting its role in tumor proliferation [62].

MMP-14, a membrane-type MMP, is also central to tumor invasion, as it activates other MMPs, such as MMP-2, on the cell surface, further enhancing pericellular matrix degradation and enabling cells’ infiltration in GBM [46]. Another study suggested that elevated protein levels of MMP-13 in GBM patients were correlated with the aggressive and invasive nature of these tumors [63]. Metastatic cell adhesion to the brain capillary endothelium may involve MMPs, although their precise role in this phase of metastasis remains uncertain. Given their diverse functions, MMPs likely facilitate this process. Hummel et al. demonstrated that MMP-3 and MMP-12 facilitate the shedding of adhesion molecules like vascular cell adhesion molecule-1 (VCAM-1) and intercellular cell adhesion molecule-1 (ICAM-1) from human brain endothelial cell membranes after MMP upregulation induced by TNF-α [64]. The study by Nakada et al. highlighted the roles of MT1-MMP and MT2-MMP in glioblastomas, showing their elevated expression compared to lower-grade astrocytomas and normal brain tissue. These MMPs synergistically activate pro-MMP-2, promoting ECM degradation and tumor invasion. Immunohistochemistry localized MT1-MMP and MT2-MMP predominantly in neoplastic astrocytes and endothelial cells. In contrast, MT3-MMP showed minimal expression and no correlation with pro-MMP-2 activation, emphasizing the critical roles of MT1-MMP and MT2-MMP in GBM progression [65].

### 3.2. Angiogenesis

MMPs also play an essential role in angiogenesis, which is necessary to support the growth and survival of rapidly proliferating tumor cells [36]. In brain tumors, angiogenesis is markedly upregulated, leading to the formation of an abnormal, disorganized vasculature that sustains tumor growth by supplying essential nutrients and oxygen within the restricted intracranial space [66]. MMP-2 and MMP-9 are particularly involved in the angiogenesis of human gliomas, as their degradation of ECM components releases stored pro-angiogenic factors, such as VEGF [60]. This process not only supports tumor growth but also contributes to the creation of an abnormal vascular network, which exacerbates tumor hypoxia, further driving MMPs’ expression and perpetuating a cycle that sustains tumor progression [67]. Immunohistochemistry revealed MMP-2 positivity in 38% of glioma cases and MMP-9 positivity in 81%, with MMP-9 also observed in endothelial cells, suggesting its role in angiogenesis. Quantitative RT-PCR showed elevated MMP-2 mRNA levels in cases with recurrence or dissemination (*p* < 0.05) and MMP-9 mRNA levels that were significantly higher in GBM (*p* < 0.05). The Ki-67 proliferation index increased with the tumor grade, correlating with recurrence and dissemination. These findings indicate that MMP-2 and MMP-9 expression, alongside Ki-67, may serve as a biomarker for glioma invasiveness, recurrence, and malignancy [68].

An immunocytochemical analysis revealed the strong expression of MMP-3 and MMP-10, particularly in the ECM-surrounding blood vessels and over 90% of neoplastic cells, with a high staining intensity. These findings suggest that MMP-3 and MMP-10 play pivotal roles in the pathogenesis of pediatric astrocytomas, emphasizing their potential contributions to tumor invasion and ECM remodeling [69].

### 3.3. Immune Evasion and Modulation

In addition to facilitating invasion and angiogenesis, MMPs are implicated in immune evasion within the brain tumor microenvironment, and tumor cells often manipulate MMPs to modulate immune responses, creating an environment that suppresses immune cell activity and protects the tumor from immune detection [70]. MMPs can process and release immunosuppressive cytokines and chemokines, which alter immune cell recruitment and function, thereby promoting an immunosuppressive milieu. For instance, MMP-7 and MMP-9 have been shown to influence the availability of cytokines like interleukin-6 (IL-6) and tumor necrosis factor-alpha (TNF-α), modulating inflammatory signaling and dampening immune cell responses in the tumor microenvironment [71,72]. Additionally, the MMP-mediated remodeling of the ECM can affect the migration and activation of immune cells, further aiding immune evasion. This immunosuppressive environment is especially detrimental in gliomas, where the immune system is already limited in its access to the CNS, and, by reshaping the immune landscapes of brain tumors, MMPs enable cancer cells to proliferate and invade largely unchecked by the body’s immune defenses [73].

Beyond general immune modulation, MMPs specifically influence the glioma immune microenvironment through direct and indirect interactions with immune checkpoint molecules and immune cell infiltration [74]. MMP-9, for example, can modulate immune responses by cleaving immune checkpoint molecule ligands such as PD-L1, altering their surface expression and affecting immune cell interactions [75]. Furthermore, MMP activity reshapes glioma-associated immune cell profiles, notably promoting tumor-associated macrophage (TAM) infiltration and polarization towards immunosuppressive M2 phenotypes, thereby dampening antitumor immune responses [76]. Additionally, MMPs, including MMP-2 and MMP-14, facilitate the recruitment and migration of regulatory T-cells (Tregs) and myeloid-derived suppressor cells (MDSCs) into glioma tissue by remodeling extracellular matrix components and modulating chemokine gradients [77]. Collectively, these mechanisms underscore MMPs’ roles in promoting immunosuppression within glioma microenvironments, suggesting that their therapeutic targeting could enhance the efficacy of immunotherapies.

### 3.4. Microenvironmental Remodeling

Another significant role of MMPs in glioma progression is their involvement in remodeling the tumor microenvironment. The brain tumor microenvironment is characterized by a complex interaction among tumor cells, stromal cells, ECM components, and signaling molecules, which together create a supportive niche for cancer cell survival and growth. Through their enzymatic activities, MMPs promote this microenvironmental remodeling by modifying the ECM’s stiffness and composition, which influences cancer cell behavior, migration, and the response to therapy. For example, MMP-11 and MMP-19 have been observed to be upregulated in certain high-grade gliomas and are thought to participate in altering the physical properties of the ECM, enabling cancer cells to migrate more freely [43]. By degrading ECM components and releasing matrix-bound growth factors, MMPs help to establish a microenvironment that supports cell proliferation, promotes stem-like cancer cell properties, and enhances the resistance to conventional therapies, and this remodeling process is dynamic and adapts to the changing needs of the tumor, underscoring the complexity of MMPs’ roles in tumor biology [78].

### 3.5. Disruption of the Blood–Brain Barrier (BBB)

The role of MMPs in disrupting the BBB is a crucial factor in glioma progression [79]. The BBB serves as a selective barrier that regulates the passage of substances between the bloodstream and the brain, providing a protective environment for neural tissue [80]. In brain tumors, however, MMP activity compromises BBB integrity, allowing cancer cells, immune cells, and therapeutic agents to cross more readily. While BBB disruption can potentially facilitate drug delivery, it also permits the invasion of peripheral immune cells and leads to edema, exacerbating tumor-induced inflammation and swelling [56]. MMP-2 [81], MMP-9 [82,83], and MMP-14 [84] are particularly implicated in BBB degradation in gliomas, where their activity weakens tight junctions between endothelial cells and facilitates tumor invasion into the brain parenchyma. This compromise of the BBB not only aids tumor progression but also complicates therapeutic strategies, as drug penetration across the BBB remains unpredictable and limited. Elevated levels of MT5-MMP (MMP-24) and MT6-MMP (MMP-25) have been observed in human brain tumors compared to normal brain tissue; however, further detailed analysis is needed [85,86].

Together, these roles of MMPs illustrate their multifaceted contributions to glioma progression, from local tissue invasion and angiogenesis to immune evasion, microenvironmental remodeling, and BBB disruption. The cumulative effects of these activities support an aggressive cancer phenotype that is resistant to conventional treatments. As such, MMPs represent not only potential biomarkers for the assessment of tumor aggressiveness and prognosis but also promising therapeutic targets [11]. By inhibiting specific MMPs or modulating their activity, there is potential to curb brain tumor progression, improve the efficacy of existing therapies, and ultimately enhance patient outcomes [87].

## 4. Prognostic and Diagnostic Relevance of MMPs in Gliomas

MMPs hold significant potential as both diagnostic biomarkers and prognostic indicators in gliomas, particularly gliomas where MMPs’ expression levels are often correlated with the tumor aggressiveness, grade, and patient survival outcomes [88]. As MMPs are involved in critical processes that drive brain tumor progression, including tissue invasion, angiogenesis, and immune modulation, monitoring their activity and expression levels could provide valuable information in assessing tumor behavior, predicting clinical outcomes, and guiding treatment strategies [89]. This section discusses the diagnostic and prognostic relevance of MMPs in gliomas, exploring their potential utility in early detection, tumor grading, and outcome prediction.

The expression and activity of MMPs, particularly MMP-2 [90] and MMP-9, are consistently elevated in aggressive gliomas, such as GBM, the most malignant form of glioma [91]. Studies have shown that high levels of MMP-2 [13,92] and MMP-9 [93] correlate with poor patient prognoses, greater tumor invasiveness, and shorter survival times. The gelatinases, MMP-2 and MMP-9, are thus of particular interest as biomarkers in assessing the progression of gliomas. Elevated MMP-2 [5,94] and MMP-9 [95] levels in patient tissue samples, serum, or cerebrospinal fluid (CSF) have been associated with advanced tumor grades, an increased risk of recurrence, and lower overall survival. This correlation suggests that measuring these MMPs could help to distinguish between low-grade and high-grade gliomas, allowing for more accurate prognosis and patient stratification. An analysis of the mRNA expression profiles from multiple datasets revealed that MMP-9 expression was correlated significantly with the glioma grade and survival outcomes. Low MMP-9 expression was associated with better overall survival (OS) and progression-free survival (PFS), independently of the MGMT methylation status [96].

For instance, MMP-1 has been found to correlate with enhanced invasion and metastatic potential in both primary brain tumors and brain metastases from other cancers, such as melanoma and breast cancer [97]. Elevated MMP-1 expression has been associated with increased aggressiveness and poorer outcomes in patients, suggesting its utility as a potential biomarker supporting the diagnosis of brain gliomas [15]. Similarly, MMP-7, known for its ability to modulate immune responses and ECM interactions, has been observed at high levels in various brain tumors [98]. Its expression is linked to increased tumor cell proliferation and invasiveness, underscoring its potential as a prognostic marker, particularly in metastatic gliomas [40]. MMP-14, a membrane-type MMP, is another enzyme with significant prognostic implications; its elevated levels are often associated with more invasive phenotypes, increased brain tumor grades, and poor prognoses [46]. Given its role in activating other MMPs and facilitating pericellular ECM degradation, MMP-14 is a key player in invasive brain tumors and may serve as a valuable marker for the prediction of brain tumor aggressiveness [99].

The diagnostic relevance of MMPs in gliomas also extends to their potential as biomarkers for early detection, and MMPs can be detected in various biological fluids, including blood, CSF, and serum, making them accessible for non-invasive diagnostic testing [100]. The serum levels of MMP-9, for example, have been studied in the context of gliomas and have shown promise as a diagnostic marker that could differentiate between malignant and benign brain lesions. Elevated MMP-9 levels in the CSF of patients with recurrent glioma undergoing doxycycline treatment indicated a lack of therapeutic response [95]. Similarly, the serum MMP-9 levels, assessed via ELISA, were found to increase in patients with high-grade gliomas following tumor resection, likely reflecting heightened inflammation triggered by surgery, as MMP-9 is known to rise during inflammatory processes [101,102]. Additionally, patients with GBM and radiographically detectable disease exhibited significantly higher serum MMP-9 levels compared to those without visible disease [101]. These findings suggest that MMP-9 holds promise as a biomarker for GBM progression, although further studies are essential to validate its utility. The ability to measure MMPs in serum or CSF provides an advantage, as gliomas often lack specific biomarkers that can be easily detected outside of tissue biopsies [103]. In addition to aiding in diagnosis, measuring MMPs in patient serum or CSF could allow for the real-time monitoring of disease progression and the response to therapy, which would be valuable for both initial diagnosis and ongoing management [104].

Urine samples from brain tumor patients showed significant elevations in MMP-2, MMP-9, MMP-9/NGAL, and VEGF compared to controls (all *p* < 0.001), with MMP-2 and VEGF providing the best diagnostic accuracy when combined. Immunohistochemistry confirmed the presence of these proteins in tumor tissue, and the biomarker levels decreased following tumor resection in some patients. These findings demonstrate that urinary MMPs and VEGF can predict brain tumors, offering a novel, noninvasive approach to tumor detection and monitoring [105]. Another promising application of MMPs as diagnostic markers lies in their potential to serve as indicators of BBB integrity. Since MMPs are known to disrupt the BBB by degrading basement membrane components, elevated levels of specific MMPs in the CSF or blood may indicate BBB breakdown, a process that is commonly associated with advanced brain tumors [106]. Monitoring MMPs’ levels in conjunction with imaging techniques could provide insights into the extent of BBB disruption, allowing clinicians to better assess tumor progression and evaluate the risks associated with BBB permeability [107].

Despite the promise of MMPs as diagnostic and prognostic biomarkers, several challenges remain. One major issue is the lack of standardization in MMP measurement techniques, as MMPs’ levels can vary significantly depending on the sample type, collection methods, and analytical procedures. Furthermore, MMP expression is influenced by factors such as inflammation and tissue remodeling, which can occur in response to both cancer and non-cancerous conditions, potentially confounding the interpretation of MMPs’ levels. Another challenge is that MMPs often exist in different isoforms and are subject to regulation by TIMPs, which can affect their activity and stability. Thus, the diagnostic and prognostic use of MMPs will likely require a combination of multiple biomarkers or a comprehensive panel of MMPs and TIMPs to achieve accuracy and reliability. The prognostic and diagnostic relevance of MMPs in gliomas is substantial, with several MMPs showing potential as markers for tumor aggressiveness, patient outcomes, and BBB integrity. MMP-2, MMP-9, MMP-1, MMP-7, and MMP-14, among others, stand out for their association with high-grade tumors and poor prognosis, making them valuable candidates for further investigation as clinical biomarkers. While further research is needed to address the challenges in MMP measurement and interpretation, the integration of MMP profiling into diagnostic and prognostic frameworks could enhance clinical decision-making and provide more personalized treatment approaches for glioma patients.

Variability in assay methods, including differences in sample handling, detection platforms, and quantification standards, significantly affects the reliability and comparability of MMP measurements across studies. Additionally, confounding factors such as inflammation, tissue remodeling, and concomitant pathologies can alter MMPs’ expression independently of the tumor biology, complicating the interpretation of the results. To overcome these issues, recent efforts have focused on standardizing MMP assays across laboratories through harmonized protocols and international guidelines [108]. Moreover, the validation of comprehensive MMP panels in large multicenter cohorts is underway, aiming to refine their clinical utility. Emerging liquid biopsy platforms, particularly the detection of MMPs in extracellular vesicles, show promise as minimally invasive approaches that can circumvent some of these technical challenges [109,110]. EV-based assays may offer greater specificity and robustness, enabling the real-time monitoring of glioma progression and therapeutic responses [111]. The continued development and validation of these advanced methodologies will be crucial in integrating MMP profiling into routine clinical practice.

## 5. Therapeutic Targeting of MMPs in Gliomas

MMPs have garnered significant interest as therapeutic targets in gliomas, particularly in high-grade gliomas, due to their roles in tumor invasion, angiogenesis, immune modulation, and BBB disruption. MMPs facilitate the breakdown of ECM components, enabling cancer cell infiltration into adjacent brain tissue, which poses a substantial challenge for complete tumor resection and results in a poor prognosis [112]. Targeting MMPs could therefore provide a multifaceted approach that could limit tumor growth, reduce its invasiveness, and potentially improve patient outcomes by combining MMP inhibition with standard therapies [113]. Additionally, recent computational studies have demonstrated the utility of techniques such as molecular docking and QSAR modeling in identifying novel cancer biomarkers (e.g., MMP) inhibitors, offering promising avenues for therapeutic advancements in gliomas and other types of cancer [114,115]. This section reviews recent strategies for the targeting of MMPs in gliomas, highlighting the types of MMP inhibitors, the molecular mechanisms that they exploit, the preclinical and clinical outcomes, and the challenges faced in translating these approaches into effective treatments.

### 5.1. Broad-Spectrum MMP Inhibitors

The development of small-molecule inhibitors targeting specific MMPs has been a focus of therapeutic research. Early inhibitors, including broad-spectrum MMP inhibitors such as marimastat, showed promise in blocking MMP activity, but they also led to adverse side effects due to the inhibition of multiple MMPs, some of which play beneficial roles in normal tissue maintenance [116]. Thus, research has increasingly focused on designing selective inhibitors that target specific MMPs associated with tumor progression, notably MMP-2 [117], MMP-9 [118], and MMP-14 [119].

A study explored the role of MMP-2 in GBM, focusing on its effects on tumor progression, angiogenesis, and invasion, using a genetically engineered mouse model [90]. MMP-2, expressed in both tumor and host cells, was shown to regulate vascular patterning and tumor cell behavior in a dose-dependent manner. The complete absence of MMP-2 led to a paradoxical increase in vascular density characterized by highly branched, non-functional blood vessels with poor perfusion. This vascular phenotype was associated with increased tumor cell apoptosis and prolonged survival in tumor-bearing mice, indicating that MMP-2 is essential for functional angiogenesis in GBM. Interestingly, while MMP-2 deficiency reduced tumor infiltration into brain parenchyma, it promoted perivascular invasion, with tumor cells migrating along blood vessels. Additionally, the absence of MMP-2 impaired pericyte recruitment and activation, further contributing to vascular dysfunction [90]. Another study demonstrated that glioma invasion was driven by MMP-2 activity, which was significantly higher in glioma cell lines than in non-invasive glial cells. MMP-2 activity was strongly correlated with tumor invasiveness (r^2^ = 0.95) and could be inhibited by metalloprotease inhibitors or protein kinase C (PKC) inhibition via calphostin C, reducing invasion by >90%. The activation of PKC in non-invasive astrocytes increased their MMP-2 activity and invasiveness, highlighting PKC’s regulatory role in glioma invasion. These findings establish MMP-2 as a critical effector of glioma invasion modulated by PKC [120].

The study by Li et al. identified preoptic regulatory factor-2 (Porf-2) as a suppressor of tumor migration in neuroblastoma and glioma. An analysis of tumor databases and cell lines revealed that Porf-2 expression is reduced in these malignancies. Functional assays demonstrated that Porf-2 inhibits tumor cell migration through its GAP domain, which inactivates Rac1. Additionally, Porf-2 downregulates MMP-2 and MMP-9, key mediators of extracellular matrix degradation. The overexpression of MMP-2 reversed the inhibitory effect of Porf-2 on tumor migration in vitro and in vivo. These findings establish Porf-2 as a regulator of tumor migration and suggest its therapeutic potential in nervous system tumors [121]. Further studies on MMP-2 inhibitors, such as the selective gelatinase inhibitor SB-3CT, revealed a reduction in glioma cell invasion and a decrease in angiogenesis by preventing endothelial cell migration, a critical process in tumor vascularization. These results underscore the importance of MMP-2 as a target in limiting both tumor spread and its supportive vascular network [122,123].

MMP-9, another gelatinase, is highly expressed in aggressive gliomas and contributes to invasion, immune suppression, and resistance to therapy. MMP-9 inhibition has shown significant potential in both reducing the invasiveness of tumor cells and countering immune suppression within the tumor microenvironment. GBM patients with low MMP-9 expression showed improved responses to temozolomide (TMZ) chemotherapy. These findings suggest that MMP-9 may serve as a critical biomarker for prognosis and therapeutic responses in GBM patients [96]. Another study revealed that TMZ treatment in GBM cells induces the upregulation of MMP-9, potentially promoting tumor invasion and a poor prognosis. While TMZ effectively killed GBM cells at 500 µM, surviving cells exhibited increased MMP-9 expression and activity, without similar changes in MMP-2. MMP-9 upregulation was mediated through p38 and JNK phosphorylation, which activated AP-1 by upregulating c-Fos and c-Jun. Subcutaneous tumors post-TMZ treatment also showed elevated MMP-9 levels and gelatinolytic activity. Inhibition of the p38 or JNK pathways counteracted these effects. These findings highlight a potential adverse effect of TMZ treatment via increased MMP-9 expression and underscore the need to address this mechanism in therapeutic strategies [124]. Dibdiakova et al. demonstrated that elevated MMP-9 levels were predictive of an aggressive GBM phenotype and correlated with resistance to conventional therapies, indicating that MMP-9 is a relevant therapeutic target not only in halting tumor progression but also in potentially sensitizing tumors to existing treatments. The recent development of highly specific MMP-9 inhibitors, such as those based on modified peptide sequences that bind directly to the MMP-9 active site, has shown promise in preclinical models by minimizing the invasive capacity and supporting immune cell activity within the tumor microenvironment [63].

In the study by Zhong et al., they evaluated the effects of norepinephrine on GBM migration and invasion, focusing on its role in regulating MMP-11. Norepinephrine treatment significantly suppressed migration and invasion in U87 and U251 GBM cells, with a corresponding decrease in MMP-11 expression, as revealed by the Human Tumor Metastasis RT2 Profiler PCR Array. Silencing MMP-11 similarly reduced cell migration and invasion, while β-adrenergic receptor (β-AR) blockade with propranolol reversed norepinephrine’s effects in U251 cells but not in U87 cells. Additionally, GBM patients with higher MMP-11 expression exhibited poorer prognoses. These findings suggest that norepinephrine inhibits GBM progression, potentially via MMP-11 downregulation, with β-AR playing a cell-line-specific regulatory role [125]. Ginsenoside Rh2 (GRh2) significantly reduced GBM cell migration and invasiveness in a dose-dependent manner, as shown in scratch wound healing and Transwell assays. The suppression of cell migration was linked to the decreased expression of MMP-13, mediated through the inhibition of the PI3K/Akt signaling pathway. High levels of MMP-13 were observed in GBM patient specimens, reinforcing its role in tumor invasiveness. These findings reveal a novel function of GRh2 in reducing GBM metastasis by targeting Akt-mediated MMP-13 activation, suggesting its potential as a therapeutic agent for invasive GBM [126].

MMP-14 or MT1-MMP is also a promising target due to its role in local ECM degradation and in activating other MMPs, including MMP-2, on the tumor cell surface [9]. An analysis of glioma tissue from 55 patients revealed that lower Tip60 (histone acetyltransferase Tat-interacting protein 60 kDa) expression was correlated with higher tumor grades and increased MT1-MMP expression. In GBM cells, Tip60 knockdown enhanced cell adhesion, spreading, and MT1-MMP transcription, promoting invasion. These effects were suppressed by inhibiting MT1-MMP and the NF-κB pathway. This study identifies Tip60 as a regulator of GBM invasiveness, demonstrating its role in downregulating MT1-MMP expression and cell adhesion through NF-κB suppression, thus highlighting Tip60 as a potential therapeutic target [127].

Several broad-spectrum MMP inhibitors, such as marimastat and batimastat, entered clinical trials, with high expectations based on their preclinical success. However, these trials failed, primarily due to poor pharmacokinetics, a lack of tumor specificity, and significant off-target toxicity, especially musculoskeletal pain and inflammation [128]. These outcomes underscored the importance of isoform selectivity and localized delivery in future drug development. To overcome these challenges, current strategies emphasize precision inhibition through isoform-specific small molecules [116], TIMP mimetics [26,129], antibodies [130,131], and targeted delivery platforms such as nanoparticles [132,133] and antibody conjugates [134], which aim to restrict the MMP blockade to the tumor microenvironment and reduce systemic side effects.

### 5.2. Natural Compounds as MMP Inhibitors

Beyond synthetic inhibitors, natural compounds with MMP-inhibitory properties are under investigation for their potential in glioma therapy. For example, certain polyphenols in green tea, such as epigallocatechin gallate (EGCG), have been shown to inhibit MMP-9 expression, reducing glioma cells’ invasiveness and angiogenesis in vitro. These natural compounds exhibit relatively low toxicity and have synergistic effects when combined with other therapies. Flavonoids, found in fruits and vegetables, have also demonstrated MMP-inhibitory effects in glioma cell lines, potentially offering a complementary approach to managing gliomas [135,136].

### 5.3. Gene Therapy Approaches Targeting MMPs

Gene therapy has emerged as a novel strategy to modulate MMPs’ expression in gliomas, particularly by using gene silencing techniques such as RNA interference (RNAi) to knock down MMP-2 or MMP-9 expression. Preclinical studies using siRNA or shRNA targeting MMP-2 or MMP-9 have shown that reducing these MMPs’ expression diminishes glioma cell invasion and enhances the response to chemotherapeutic agents [137,138]. Zhao et al. investigated the effects of the volatile anesthetic sevoflurane on glioma cell migration and invasion. Sevoflurane was found to suppress migration and invasion in glioma cells (LN229 and U251) by upregulating microRNA-34a-5p (miR-34a-5p) and downregulating MMP-2. Mechanistically, miR-34a-5p directly targets MMP-2, and its overexpression enhanced the sevoflurane-induced inhibition of migration and invasion, while its knockdown reversed these effects. MMP-2 silencing compensated for the loss of miR-34a-5p under sevoflurane. These findings reveal a novel mechanism whereby sevoflurane inhibits glioma cell migration and invasion via the miR-34a-5p/MMP-2 axis, offering insights into its pharmacological impact during glioma surgery [139].

Another study demonstrated that MT1-MMP plays a critical role in GBM radio-resistance and tumor invasiveness. An analysis of patient data revealed that MT1-MMP expression was inversely correlated with survival, and its activity was confirmed in GBM brain tissue but absent in normal brains. Using glioma stem-like neurospheres (GSCs), the study showed that MT1-MMP inhibition, via shRNA knockdown or the brain-permeable inhibitor (R)-ND336, sensitized cells to radiation, increased double-strand breaks (DSBs), and enhanced cytotoxic responses. Additionally, MT1-MMP inhibition reduced invasion by modulating MMP-2 activity. In vivo, (R)-ND336 significantly improved tumor control and survival. These findings identify MT1-MMP as a promising therapeutic target and (R)-ND336 as a novel radiosensitizer for GBM treatment [140].

MMP-9-targeted siRNA in a GBM mouse model led to decreased tumor invasion and improved survival compared to untreated controls. Another approach involves using viral vectors to deliver genes encoding inhibitors of specific MMPs directly to tumor cells, effectively reducing MMPs’ activity at the tumor site. While these gene therapy approaches show promise, challenges remain, particularly in delivering gene silencing agents across the BBB. Non-viral delivery methods, such as nanoparticles and liposomes, are currently under investigation to enhance the delivery of MMP-targeted RNAi agents to brain tumors. These methods could potentially improve the specificity and efficacy of MMP inhibition in gliomas, with minimal systemic toxicity [141,142,143].

### 5.4. Emerging Delivery Systems: Nanoparticles and TIMP Mimetics

To further overcome the challenges related to BBB permeability and systemic toxicity, innovative strategies are currently being explored. One promising approach is nanoparticle-mediated delivery systems, including polymeric nanoparticles, liposomes, and dendrimers, which can selectively transport MMP inhibitors directly across the BBB to glioma tissue [144]. These nanocarriers offer targeted, controlled release, significantly enhancing the therapeutic index by increasing drug accumulation specifically at tumor sites and minimizing off-target effects [145]. Another emerging approach involves the use of engineered TIMP mimetics or TIMP-based fusion proteins, which provide the selective inhibition of pathological MMP isoforms implicated in glioma progression [129]. TIMPs are critical regulators of MMPs and play a complex role in glioma progression [63]. TIMPs exhibit distinct regulatory mechanisms and biological effects. For example, TIMP-1 has been shown to promote cell survival and proliferation through interactions with β1 integrins and CD63, independently of its MMP-inhibitory activity [146]. Conversely, TIMP-3 is tightly bound to the ECM and exhibits anti-tumorigenic properties by inducing apoptosis, inhibiting angiogenesis, and suppressing tumor cell invasion [147]. Interestingly, TIMP-2 demonstrates a dual role: at lower concentrations, it facilitates MMP-2 activation by acting as a bridge between MT1-MMP and pro-MMP-2; however, at higher concentrations, it inhibits MMP-2 activity, thereby reducing glioma invasiveness [148]. The dysregulated expression of TIMPs has been observed in gliomas, with studies reporting reduced TIMP-1 and -2 levels correlating with higher tumor grades [149]. These findings highlight the critical balance between MMPs and TIMPs in determining glioma progression and suggest that modulating TIMP activity could be a promising therapeutic strategy. Such TIMP mimetics leverage natural regulatory mechanisms and can be designed for improved stability, selectivity, and BBB penetration. These innovative delivery and targeting approaches hold significant promise in advancing the clinical translation and therapeutic effectiveness of MMP-targeted treatments in gliomas.

### 5.5. Challenges and Future Directions

Although MMP-targeted therapies hold great promise, several challenges must be addressed to transfer these approaches to clinical settings. One major obstacle is the dual role of MMPs in both normal tissue maintenance and pathological processes; thus, MMP inhibition can lead to off-target effects, including compromised wound healing, altered immune responses, and joint stiffness, as observed with earlier broad-spectrum MMP inhibitors [150]. The selective inhibition of specific MMPs, or the targeted delivery of inhibitors directly to the tumor site, is therefore essential to minimize such side effects. Moreover, the complex interplay between MMPs and TIMPs adds another layer of regulatory complexity, where the balance between MMP-9 and TIMP-1 has been shown to influence glioma invasiveness, with higher TIMP-1 levels correlating with reduced MMP-9 activity and, consequently, reduced tumor invasion [151]. Future therapies may therefore need to consider co-modulating TIMPs to optimize MMP inhibition selectively within the tumor microenvironment. New research on engineered TIMP mimetics or TIMP fusion proteins could offer more precise control over MMPs’ activity.

Combination therapies integrating MMP inhibitors with existing cancer treatments such as chemotherapy and radiotherapy could also enhance the efficacy of MMP-targeted therapies [152]. By weakening the ECM structure and reducing tumor cell migration, MMP inhibition could make cancer cells more vulnerable to chemotherapeutic agents. Recent studies have shown that MMP-2 and MMP-9 inhibition can enhance the penetration of chemotherapy drugs across the BBB, potentially improving their efficacy in treating brain tumors [61,153]. Furthermore, combining MMP inhibition with anti-angiogenic therapies may restrict the tumor’s blood supply while limiting its invasive capacity, creating a dual attack on tumor growth and spread [154]. Lastly, ongoing research on the role of MMPs in immune modulation suggests that MMP inhibition may also have synergistic effects with immunotherapy. Since MMPs can degrade cytokines and chemokines, thereby creating an immunosuppressive environment, inhibiting MMPs might enhance immune cell infiltration and activity within the tumor [10,155].

Historically, the clinical translation of MMP inhibitors has faced significant challenges. Broad-spectrum MMP inhibitors, such as marimastat, initially showed promise in preclinical studies but ultimately failed in clinical trials due to significant off-target effects, including musculoskeletal side effects, and a lack of clinical efficacy, stemming from their non-selective inhibition of beneficial MMPs involved in normal physiological processes. This highlighted the need for greater selectivity in inhibitor design. Recent advances have shifted the focus toward isoform-selective inhibitors targeting specific MMPs that are critical to glioma progression, notably MMP-9 and MMP-14. These selective inhibitors have demonstrated improved efficacy and reduced systemic toxicity in preclinical glioma models. For instance, selective MMP-9 inhibitors have shown potential in limiting tumor invasiveness and enhancing therapeutic sensitivity [156], while MMP-14 (MT1-MMP)-specific inhibitors have effectively restricted tumor invasion and improved the response to radiation therapy [157]. The current clinical development and ongoing trials are focused on validating these isoform-selective inhibitors, underscoring the importance of targeted approaches to overcome the limitations observed with earlier broad-spectrum inhibitors.

## 6. Emerging Research and Future Directions

The understanding of MMPs in gliomas is evolving rapidly, with research increasingly focusing on innovative therapeutic approaches, advanced biomarker applications, and new insights into MMP-related mechanisms in brain tumors. Recent technological advances in bioinformatics, genomics, and proteomics are uncovering previously unrecognized roles of MMPs in gliomas and providing novel approaches for MMP-targeted interventions. One promising area of research is the use of high-throughput bioinformatics and gene expression databases to identify MMP expression patterns specific to GBM and other aggressive brain tumors [158]. For example, bioinformatics analyses have shown that certain MMPs, such as MMP-1, MMP-11, MMP-19, and MMP-21, are consistently upregulated in GBM compared to normal brain tissue, suggesting that they may serve as biomarkers for diagnosis and prognosis in high-grade gliomas [91]. Additionally, multiomics analyses integrating transcriptomics, proteomics, and methylomics have revealed potential regulatory mechanisms controlling MMP expression, including epigenetic modifications and signaling pathway interactions that are specific to the tumor microenvironment [159,160].

Another frontier in MMP research involves exploring MMPs’ roles in immune modulation within the tumor microenvironment. Research has increasingly shown that MMPs, particularly MMP-2 and MMP-9, influence immune cell trafficking, cytokine release, and immune suppression in brain tumors, potentially by modulating the expression and activity of immune checkpoint molecules [161,162]. This modulation of immune processes suggests that MMP inhibition could be synergized with immunotherapy, which has so far shown limited success in gliomas due to immune resistance and the BBB. For instance, targeting MMP-9 in conjunction with immune checkpoint inhibitors could potentially enhance immune cell infiltration and activity within glioma tumors [122].

New research is also being conducted on delivery systems capable of transporting MMP inhibitors across the BBB, which is a major hurdle for glioma treatment, and advances in nanoparticle-based delivery systems, such as liposomes, polymeric nanoparticles, and dendrimers, have shown promise for the targeted delivery of MMP inhibitors to tumor sites, allowing higher drug concentrations at the tumor while reducing systemic toxicity [163]. Additionally, functionalized nanoparticles that respond to the tumor microenvironment by releasing their payloads in response to specific triggers, such as pH changes or enzymatic activity, are currently under investigation and may offer even greater specificity and efficacy for MMP-targeted therapies in brain tumors [164].

Another intriguing avenue for MMP-related research is the use of extracellular vesicles as both biomarkers and delivery systems, where studies have demonstrated that MMPs are present in serum-derived EVs, which can cross the BBB and contain information reflective of the tumor’s molecular characteristics [165]. The MMP-9 levels in extracellular vesicles have already shown promise as biomarkers for GBM and may allow for the non-invasive monitoring of disease progression and the treatment response. Moreover, EVs can potentially be engineered to carry MMP inhibitors or RNA molecules designed to silence MMP expression, offering a dual diagnostic and therapeutic function [14].

The development of selective inhibitors and TIMP-based therapies represents another exciting direction. Although broad-spectrum MMP inhibitors had significant side effects in earlier trials, the identification of MMP isoforms that drive specific pathways in gliomas has opened the door to more targeted treatments [26]. TIMP analogs engineered to inhibit specific MMPs have shown promise in preclinical studies and could provide a more selective approach to MMP inhibition that minimizes off-target effects [166]. Additionally, TIMP fusion proteins, designed to deliver TIMP molecules directly to tumor sites, are currently being tested and may offer a novel therapeutic option for the control of MMPs’ activity specifically within the tumor microenvironment [167].

Despite promising advances, several translational challenges continue to hinder the clinical implementation of MMP-targeted therapies. One major barrier is effective delivery across the BBB, which limits the penetration of therapeutic agents into the tumor site. Innovative strategies, including nanoparticle-mediated delivery systems and BBB-permeable small molecules, are currently under investigation to address this issue. Another critical challenge is tumor heterogeneity—both inter- and intra-tumoral variability in MMP expression can lead to inconsistent therapeutic responses. To overcome this, personalized treatment approaches guided by the molecular profiling of patient tumors may be necessary. Finally, achieving high specificity in MMP inhibition remains difficult, as many MMPs are involved in normal tissue remodeling. Isoform-selective inhibitors, engineered TIMP analogs, and tumor-specific targeting moieties are being developed to minimize off-target effects while maintaining efficacy.

The future of MMP research in gliomas lies in the integration of molecular insights, advanced delivery methods, and combination therapies that synergize with existing treatments. Continued investigation into MMPs’ roles in tumor–immune interactions, innovative delivery platforms, and non-invasive biomarkers is likely to yield new strategies that improve both the specificity and efficacy of MMP-targeted therapies.

## References

[1] Nabors L.B., Ammirati M., Bierman P.J., Brem H., Butowski N., Chamberlain M.C., DeAngelis L.M., Fenstermaker R.A., Friedman A., Gilbert M.R. (2013). Central nervous system cancers. J. Natl. Compr. Cancer Netw..

[2] Louis D.N., Perry A., Wesseling P., Brat D.J., Cree I.A., Figarella-Branger D., Hawkins C., Ng H.K., Pfister S.M., Reifenberger G. (2021). The 2021 WHO Classification of Tumors of the Central Nervous System: A summary. Neuro-Oncol..

[3] Yalamarty S.S.K., Filipczak N., Li X., Subhan M.A., Parveen F., Ataide J.A., Rajmalani B.A., Torchilin V.P. (2023). Mechanisms of Resistance and Current Treatment Options for Glioblastoma Multiforme (GBM). Cancers.

[4] Hambardzumyan D., Bergers G. (2015). Glioblastoma: Defining Tumor Niches. Trends Cancer.

[5] Ganguly K., Adhikary K., Acharjee A., Acharjee P., Trigun S.K., Mutlaq A.S., Ashique S., Yasmin S., Alshahrani A.M., Ansari M.Y. (2024). Biological significance and pathophysiological role of Matrix Metalloproteinases in the Central Nervous System. Int. J. Biol. Macromol..

[6] Laronha H., Caldeira J. (2020). Structure and Function of Human Matrix Metalloproteinases. Cells.

[7] Shoari A. (2024). Potential of MMP-2 and MMP-9 Gelatinase Blockade as a Therapeutic Strategy in Fibrosarcoma Treatment: A Decadal Review. Targets.

[8] Radisky E.S. (2024). Extracellular proteolysis in cancer: Proteases, substrates, and mechanisms in tumor progression and metastasis. J. Biol. Chem..

[9] Niland S., Riscanevo A.X., Eble J.A. (2021). Matrix Metalloproteinases Shape the Tumor Microenvironment in Cancer Progression. Int. J. Mol. Sci..

[10] Wang Q., Wang K., Tan X.J., Li Z.X., Wang H.Y. (2022). Immunomodulatory role of metalloproteases in cancers: Current progress and future trends. Front. Immunol..

[11] Roy R., Yang J., Moses M.A. (2009). Matrix metalloproteinases as novel biomarkers and potential therapeutic targets in human cancer. J. Clin. Oncol..

[12] Hagemann C., Anacker J., Ernestus R.I., Vince G.H. (2012). A complete compilation of matrix metalloproteinase expression in human malignant gliomas. World J. Clin. Oncol..

[13] Sinceviciute R., Vaitkiene P., Urbanaviciute R., Steponaitis G., Tamasauskas A., Skiriute D. (2018). MMP2 is associated with glioma malignancy and patient outcome. Int. J. Clin. Exp. Pathol..

[14] Dobra G., Gyukity-Sebestyen E., Bukva M., Harmati M., Nagy V., Szabo Z., Pankotai T., Klekner A., Buzas K. (2023). MMP-9 as Prognostic Marker for Brain Tumours: A Comparative Study on Serum-Derived Small Extracellular Vesicles. Cancers.

[15] Oldak L., Chludzinska-Kasperuk S., Milewska P., Grubczak K., Reszec J., Gorodkiewicz E. (2022). MMP-1, UCH-L1, and 20S Proteasome as Potential Biomarkers Supporting the Diagnosis of Brain Glioma. Biomolecules.

[16] Dimitrova I., Tacheva T., Mindov I., Petrov B., Aleksandrova E., Valkanov S., Gulubova M., Vlaykova T. (2019). Serum levels of MMP-7 in primary brain cancers and brain metastases. Biotechnol. Biotechnol. Equip..

[17] Gonzalez-Avila G., Sommer B., Mendoza-Posada D.A., Ramos C., Garcia-Hernandez A.A., Falfan-Valencia R. (2019). Corrigendum to “Matrix metalloproteinases participation in the metastatic process and their diagnostic and therapeutic applications in cancer” [Crit. Rev. Oncol. Hematol. 137, May (2019) 57–83]. Crit. Rev. Oncol./Hematol..

[18] Chintala S.K., Tonn J.C., Rao J.S. (1999). Matrix metalloproteinases and their biological function in human gliomas. Int. J. Dev. Neurosci..

[19] Fingleton B. (2008). MMPs as therapeutic targets—Still a viable option?. Semin. Cell Dev. Biol..

[20] Cabral-Pacheco G.A., Garza-Veloz I., Castruita-De la Rosa C., Ramirez-Acuna J.M., Perez-Romero B.A., Guerrero-Rodriguez J.F., Martinez-Avila N., Martinez-Fierro M.L. (2020). The Roles of Matrix Metalloproteinases and Their Inhibitors in Human Diseases. Int. J. Mol. Sci..

[21] Rempe R.G., Hartz A.M.S., Bauer B. (2016). Matrix metalloproteinases in the brain and blood-brain barrier: Versatile breakers and makers. J. Cereb. Blood Flow Metab..

[22] Ra H.J., Parks W.C. (2007). Control of matrix metalloproteinase catalytic activity. Matrix Biol..

[23] Cui N., Hu M., Khalil R.A. (2017). Biochemical and Biological Attributes of Matrix Metalloproteinases. Prog. Mol. Biol. Transl. Sci..

[24] Dufour A., Sampson N.S., Zucker S., Cao J. (2008). Role of the hemopexin domain of matrix metalloproteinases in cell migration. J. Cell Physiol..

[25] Itoh Y. (2015). Membrane-type matrix metalloproteinases: Their functions and regulations. Matrix Biol..

[26] Shoari A., Khalili-Tanha G., Coban M.A., Radisky E.S. (2023). Structure and computation-guided yeast surface display for the evolution of TIMP-based matrix metalloproteinase inhibitors. Front. Mol. Biosci..

[27] Amar S., Smith L., Fields G.B. (2017). Matrix metalloproteinase collagenolysis in health and disease. Biochim. Biophys. Acta (BBA)-Mol. Cell Res..

[28] Nikolov A., Popovski N. (2021). Role of Gelatinases MMP-2 and MMP-9 in Healthy and Complicated Pregnancy and Their Future Potential as Preeclampsia Biomarkers. Diagnostics.

[29] Farina A.R., Mackay A.R. (2014). Gelatinase B/MMP-9 in Tumour Pathogenesis and Progression. Cancers.

[30] Van Hove I., Lemmens K., Van de Velde S., Verslegers M., Moons L. (2012). Matrix metalloproteinase-3 in the central nervous system: A look on the bright side. J. Neurochem..

[31] Turunen S.P., Tatti-Bugaeva O., Lehti K. (2017). Membrane-type matrix metalloproteases as diverse effectors of cancer progression. Biochim. Biophys. Acta (BBA)-Mol. Cell Res..

[32] Kessenbrock K., Plaks V., Werb Z. (2010). Matrix Metalloproteinases: Regulators of the Tumor Microenvironment. Cell.

[33] Sternlicht M.D., Werb Z. (2001). How matrix metalloproteinases regulate cell behavior. Annu. Rev. Cell Dev. Biol..

[34] Nagase H., Visse R., Murphy G. (2006). Structure and function of matrix metalloproteinases and TIMPs. Cardiovasc. Res..

[35] Papaharalambus C.A., Griendling K.K. (2007). Basic mechanisms of oxidative stress and reactive oxygen species in cardiovascular injury. Trends Cardiovasc. Med..

[36] Quintero-Fabian S., Arreola R., Becerril-Villanueva E., Torres-Romero J.C., Arana-Argaez V., Lara-Riegos J., Ramirez-Camacho M.A., Alvarez-Sanchez M.E. (2019). Role of Matrix Metalloproteinases in Angiogenesis and Cancer. Front. Oncol..

[37] Henke E., Nandigama R., Ergun S. (2019). Extracellular Matrix in the Tumor Microenvironment and Its Impact on Cancer Therapy. Front. Mol. Biosci..

[38] Pullen N.A., Anand M., Cooper P.S., Fillmore H.L. (2012). Matrix metalloproteinase-1 expression enhances tumorigenicity as well as tumor-related angiogenesis and is inversely associated with TIMP-4 expression in a model of glioblastoma. J. Neurooncol.

[39] Kim E.M., Hwang O. (2011). Role of matrix metalloproteinase-3 in neurodegeneration. J. Neurochem..

[40] Rome C., Arsaut J., Taris C., Couillaud F., Loiseau H. (2007). MMP-7 (matrilysin) expression in human brain tumors. Mol. Carcinog..

[41] Lee E.J., Han J.E., Woo M.S., Shin J.A., Park E.M., Kang J.L., Moon P.G., Baek M.C., Son W.S., Ko Y.T. (2014). Matrix metalloproteinase-8 plays a pivotal role in neuroinflammation by modulating TNF-alpha activation. J. Immunol..

[42] Zhang G., Miyake M., Lawton A., Goodison S., Rosser C.J. (2014). Matrix metalloproteinase-10 promotes tumor progression through regulation of angiogenic and apoptotic pathways in cervical tumors. BMC Cancer.

[43] Stojic J., Hagemann C., Haas S., Herbold C., Kuhnel S., Gerngras S., Roggendorf W., Roosen K., Vince G.H. (2008). Expression of matrix metalloproteinases MMP-1, MMP-11 and MMP-19 is correlated with the WHO-grading of human malignant gliomas. Neurosci. Res..

[44] Li G.S., Tang Y.X., Zhang W., Li J.D., Huang H.Q., Liu J., Fu Z.W., He R.Q., Kong J.L., Zhou H.F. (2024). MMP12 is a Potential Predictive and Prognostic Biomarker of Various Cancers Including Lung Adenocarcinoma. Cancer Control.

[45] Wang J., Li Y., Wang J., Li C., Yu K., Wang Q. (2012). Increased expression of matrix metalloproteinase-13 in glioma is associated with poor overall survival of patients. Med. Oncol..

[46] Ulasov I., Yi R., Guo D., Sarvaiya P., Cobbs C. (2014). The emerging role of MMP14 in brain tumorigenesis and future therapeutics. Biochim. Biophys. Acta (BBA)-Rev. Cancer.

[47] Abraham R., Schafer J., Rothe M., Bange J., Knyazev P., Ullrich A. (2005). Identification of MMP-15 as an anti-apoptotic factor in cancer cells. J. Biol. Chem..

[48] Jiang C., Wang J., Dong C., Wei W., Li J., Li X. (2017). Membranous type matrix metalloproteinase 16 induces human prostate cancer metastasis. Oncol. Lett..

[49] Sohail A., Sun Q., Zhao H., Bernardo M.M., Cho J.A., Fridman R. (2008). MT4-(MMP17) and MT6-MMP (MMP25), A unique set of membrane-anchored matrix metalloproteinases: Properties and expression in cancer. Cancer Metastasis Rev..

[50] Lettau I., Hattermann K., Held-Feindt J., Brauer R., Sedlacek R., Mentlein R. (2010). Matrix metalloproteinase-19 is highly expressed in astroglial tumors and promotes invasion of glioma cells. J. Neuropathol. Exp. Neurol..

[51] Turk B.E., Lee D.H., Yamakoshi Y., Klingenhoff A., Reichenberger E., Wright J.T., Simmer J.P., Komisarof J.A., Cantley L.C., Bartlett J.D. (2006). MMP-20 is predominately a tooth-specific enzyme with a deep catalytic pocket that hydrolyzes type V collagen. Biochemistry.

[52] Galea C.A., Nguyen H.M., George Chandy K., Smith B.J., Norton R.S. (2014). Domain structure and function of matrix metalloprotease 23 (MMP23): Role in potassium channel trafficking. Cell. Mol. Life Sci..

[53] Llano E., Pendas A.M., Freije J.P., Nakano A., Knauper V., Murphy G., Lopez-Otin C. (1999). Identification and characterization of human MT5-MMP, a new membrane-bound activator of progelatinase a overexpressed in brain tumors. Cancer Res..

[54] Guo J.G., Guo C.C., He Z.Q., Cai X.Y., Mou Y.G. (2018). High MMP-26 expression in glioma is correlated with poor clinical outcome of patients. Oncol. Lett..

[55] Wang X., Chen X., Sun L., Bi X., He H., Chen L., Pang J. (2018). The function of MMP-28/TGF-beta induced cell apoptosis in human glioma cells. Exp. Ther. Med..

[56] Arvanitis C.D., Ferraro G.B., Jain R.K. (2020). The blood-brain barrier and blood-tumour barrier in brain tumours and metastases. Nat. Rev. Cancer.

[57] Pullen N.A., Pickford A.R., Perry M.M., Jaworski D.M., Loveson K.F., Arthur D.J., Holliday J.R., Van Meter T.E., Peckham R., Younas W. (2018). Current insights into matrix metalloproteinases and glioma progression: Transcending the degradation boundary. Met. Med..

[58] Seker-Polat F., Pinarbasi Degirmenci N., Solaroglu I., Bagci-Onder T. (2022). Tumor Cell Infiltration into the Brain in Glioblastoma: From Mechanisms to Clinical Perspectives. Cancers.

[59] Hannocks M.J., Zhang X., Gerwien H., Chashchina A., Burmeister M., Korpos E., Song J., Sorokin L. (2019). The gelatinases, MMP-2 and MMP-9, as fine tuners of neuroinflammatory processes. Matrix Biol..

[60] Wang M., Wang T., Liu S., Yoshida D., Teramoto A. (2003). The expression of matrix metalloproteinase-2 and -9 in human gliomas of different pathological grades. Brain Tumor Pathol..

[61] Yu C.F., Chen F.H., Lu M.H., Hong J.H., Chiang C.S. (2017). Dual roles of tumour cells-derived matrix metalloproteinase 2 on brain tumour growth and invasion. Br. J. Cancer.

[62] Ricci S., Guadagno E., Bruzzese D., Del Basso De Caro M., Peca C., Sgulo F.G., Maiuri F., Di Carlo A. (2017). Evaluation of matrix metalloproteinase type IV-collagenases in serum of patients with tumors of the central nervous system. J. Neuro-Oncol..

[63] Dibdiakova K., Majercikova Z., Galanda T., Richterova R., Kolarovszki B., Racay P., Hatok J. (2024). Relationship between the Expression of Matrix Metalloproteinases and Their Tissue Inhibitors in Patients with Brain Tumors. Int. J. Mol. Sci..

[64] Hummel V., Kallmann B.A., Wagner S., Fuller T., Bayas A., Tonn J.C., Benveniste E.N., Toyka K.V., Rieckmann P. (2001). Production of MMPs in human cerebral endothelial cells and their role in shedding adhesion molecules. J. Neuropathol. Exp. Neurol..

[65] Nakada M., Nakamura H., Ikeda E., Fujimoto N., Yamashita J., Sato H., Seiki M., Okada Y. (1999). Expression and tissue localization of membrane-type 1, 2, and 3 matrix metalloproteinases in human astrocytic tumors. Am. J. Pathol..

[66] Kim W.Y., Lee H.Y. (2009). Brain angiogenesis in developmental and pathological processes: Mechanism and therapeutic intervention in brain tumors. FEBS J..

[67] Winkler F., Kozin S.V., Tong R.T., Chae S.S., Booth M.F., Garkavtsev I., Xu L., Hicklin D.J., Fukumura D., di Tomaso E. (2004). Kinetics of vascular normalization by VEGFR2 blockade governs brain tumor response to radiation: Role of oxygenation, angiopoietin-1, and matrix metalloproteinases. Cancer Cell.

[68] Komatsu K., Nakanishi Y., Nemoto N., Hori T., Sawada T., Kobayashi M. (2004). Expression and quantitative analysis of matrix metalloproteinase-2 and -9 in human gliomas. Brain Tumor Pathol..

[69] Bodey B., Bodey B., Siegel S.E., Kaiser H.E. (2000). Matrix metalloproteinase expression in childhood astrocytomas. Anticancer Res..

[70] de Visser K.E., Joyce J.A. (2023). The evolving tumor microenvironment: From cancer initiation to metastatic outgrowth. Cancer Cell.

[71] Gearing A.J., Beckett P., Christodoulou M., Churchill M., Clements J., Davidson A.H., Drummond A.H., Galloway W.A., Gilbert R., Gordon J.L. (1994). Processing of tumour necrosis factor-alpha precursor by metalloproteinases. Nature.

[72] Abbes A., Zayani Y., Zidi W., Hammami M.B., Mebazaa A., El Euch D., Ben Ammar A., Sanhaji H., El May M.V., Mokni M. (2020). Matrix metalloproteinase-7 could be a predictor for acute inflammation in psoriatic patients. Cytokine.

[73] Andersen R.S., Anand A., Harwood D.S.L., Kristensen B.W. (2021). Tumor-Associated Microglia and Macrophages in the Glioblastoma Microenvironment and Their Implications for Therapy. Cancers.

[74] Sharma P., Aaroe A., Liang J., Puduvalli V.K. (2023). Tumor microenvironment in glioblastoma: Current and emerging concepts. Neuro-Oncol. Adv..

[75] Zhao F., Evans K., Xiao C., DeVito N., Theivanthiran B., Holtzhausen A., Siska P.J., Blobe G.C., Hanks B.A. (2018). Stromal Fibroblasts Mediate Anti-PD-1 Resistance via MMP-9 and Dictate TGFbeta Inhibitor Sequencing in Melanoma. Cancer Immunol. Res..

[76] Pan Y., Yu Y., Wang X., Zhang T. (2020). Tumor-Associated Macrophages in Tumor Immunity. Front. Immunol..

[77] Yuan Z.N., Li Y.P., Zhang S.F., Wang X.Y., Dou H., Yu X., Zhang Z.R., Yang S.S., Xiao M. (2023). Extracellular matrix remodeling in tumor progression and immune escape: From mechanisms to treatments. Mol. Cancer.

[78] Mustafa S., Koran S., AlOmair L. (2022). Insights Into the Role of Matrix Metalloproteinases in Cancer and its Various Therapeutic Aspects: A Review. Front. Mol. Biosci..

[79] Wrobel J.K., Toborek M. (2016). Blood-brain Barrier Remodeling during Brain Metastasis Formation. Mol. Med..

[80] Daneman R., Prat A. (2015). The blood-brain barrier. Cold Spring Harb. Perspect. Biol..

[81] Feng S., Cen J., Huang Y., Shen H., Yao L., Wang Y., Chen Z. (2011). Matrix metalloproteinase-2 and -9 secreted by leukemic cells increase the permeability of blood-brain barrier by disrupting tight junction proteins. PLoS ONE.

[82] Liu X., Su P., Meng S., Aschner M., Cao Y., Luo W., Zheng G., Liu M. (2017). Role of matrix metalloproteinase-2/9 (MMP2/9) in lead-induced changes in an in vitro blood-brain barrier model. Int. J. Biol. Sci..

[83] Mo F., Pellerino A., Soffietti R., Ruda R. (2021). Blood-Brain Barrier in Brain Tumors: Biology and Clinical Relevance. Int. J. Mol. Sci..

[84] Mohanty S., Chen Z., Li K., Morais G.R., Klockow J., Yerneni K., Pisani L., Chin F.T., Mitra S., Cheshier S. (2017). A Novel Theranostic Strategy for MMP-14-Expressing Glioblastomas Impacts Survival. Mol. Cancer Ther..

[85] Liotta L.A., Steeg P.S., Stetler-Stevenson W.G. (1991). Cancer metastasis and angiogenesis: An imbalance of positive and negative regulation. Cell.

[86] Velasco G., Cal S., Merlos-Suarez A., Ferrando A.A., Alvarez S., Nakano A., Arribas J., Lopez-Otin C. (2000). Human MT6-matrix metalloproteinase: Identification, progelatinase A activation, and expression in brain tumors. Cancer Res..

[87] Almutairi S., Kalloush H.M., Manoon N.A., Bardaweel S.K. (2023). Matrix Metalloproteinases Inhibitors in Cancer Treatment: An Updated Review (2013–2023). Molecules.

[88] Jelski W., Mroczko B. (2021). Molecular and Circulating Biomarkers of Brain Tumors. Int. J. Mol. Sci..

[89] Fares J., Fares M.Y., Khachfe H.H., Salhab H.A., Fares Y. (2020). Molecular principles of metastasis: A hallmark of cancer revisited. Signal Transduct. Target. Ther..

[90] Du R., Petritsch C., Lu K., Liu P., Haller A., Ganss R., Song H., Vandenberg S., Bergers G. (2008). Matrix metalloproteinase-2 regulates vascular patterning and growth affecting tumor cell survival and invasion in GBM. Neuro-Oncol..

[91] Karimi N., Kheiri H., Zarrinpour V., Forghanifard M.M. (2023). Bioinformatic analysis of MMP family members in GBM. Inform. Med. Unlocked.

[92] Zhang H., Ma Y., Wang H., Xu L., Yu Y. (2019). MMP-2 expression and correlation with pathology and MRI of glioma. Oncol. Lett..

[93] Choe G., Park J.K., Jouben-Steele L., Kremen T.J., Liau L.M., Vinters H.V., Cloughesy T.F., Mischel P.S. (2002). Active matrix metalloproteinase 9 expression is associated with primary glioblastoma subtype. Clin. Cancer Res..

[94] Ramachandran R.K., Sorensen M.D., Aaberg-Jessen C., Hermansen S.K., Kristensen B.W. (2017). Expression and prognostic impact of matrix metalloproteinase-2 (MMP-2) in astrocytomas. PLoS ONE.

[95] Wong E.T., Alsop D., Lee D., Tam A., Barron L., Bloom J., Gautam S., Wu J.K. (2008). Cerebrospinal fluid matrix metalloproteinase-9 increases during treatment of recurrent malignant gliomas. Cerebrospinal Fluid Res..

[96] Li Q., Chen B., Cai J., Sun Y., Wang G., Li Y., Li R., Feng Y., Han B., Li J. (2016). Comparative Analysis of Matrix Metalloproteinase Family Members Reveals That MMP9 Predicts Survival and Response to Temozolomide in Patients with Primary Glioblastoma. PLoS ONE.

[97] Liu H., Kato Y., Erzinger S.A., Kiriakova G.M., Qian Y., Palmieri D., Steeg P.S., Price J.E. (2012). The role of MMP-1 in breast cancer growth and metastasis to the brain in a xenograft model. BMC Cancer.

[98] Kaczorowska A., Miekus N., Stefanowicz J., Adamkiewicz-Drozynska E. (2020). Selected Matrix Metalloproteinases (MMP-2, MMP-7) and Their Inhibitor (TIMP-2) in Adult and Pediatric Cancer. Diagnostics.

[99] Houson H., Kasten B., Jiang K., Rao J.H., Warram J. (2019). MMP-14 as a noninvasive marker for PET and NIRF imaging of glioblastoma multiforme. J. Nucl. Med..

[100] Kan L.K., Drummond K., Hunn M., Williams D., O’Brien T.J., Monif M. (2020). Potential biomarkers and challenges in glioma diagnosis, therapy and prognosis. BMJ Neurol. Open.

[101] Hormigo A., Gu B., Karimi S., Riedel E., Panageas K.S., Edgar M.A., Tanwar M.K., Rao J.S., Fleisher M., DeAngelis L.M. (2006). YKL-40 and matrix metalloproteinase-9 as potential serum biomarkers for patients with high-grade gliomas. Clin. Cancer Res..

[102] Manicone A.M., McGuire J.K. (2008). Matrix metalloproteinases as modulators of inflammation. Semin. Cell Dev. Biol..

[103] Xiao F., Lv S., Zong Z., Wu L., Tang X., Kuang W., Zhang P., Li X., Fu J., Xiao M. (2020). Cerebrospinal fluid biomarkers for brain tumor detection: Clinical roles and current progress. Am. J. Transl. Res..

[104] Papadimitrakis D., Perdikakis M., Gargalionis A.N., Papavassiliou A.G. (2024). Biomarkers in Cerebrospinal Fluid for the Diagnosis and Monitoring of Gliomas. Biomolecules.

[105] Smith E.R., Zurakowski D., Saad A., Scott R.M., Moses M.A. (2008). Urinary biomarkers predict brain tumor presence and response to therapy. Clin. Cancer Res..

[106] Lakhan S.E., Kirchgessner A., Tepper D., Leonard A. (2013). Matrix metalloproteinases and blood-brain barrier disruption in acute ischemic stroke. Front Neurol..

[107] Harris W.J., Asselin M.C., Hinz R., Parkes L.M., Allan S., Schiessl I., Boutin H., Dickie B. (2023). In vivo methods for imaging blood-brain barrier function and dysfunction. Eur. J. Nucl. Med. Mol. I.

[108] Lopez-Avila V., Spencer J.V. (2008). Methods for detection of matrix metalloproteinases as biomarkers in cardiovascular disease. Clin. Med. Cardiol..

[109] Irmer B., Chandrabalan S., Maas L., Bleckmann A., Menck K. (2023). Extracellular Vesicles in Liquid Biopsies as Biomarkers for Solid Tumors. Cancers.

[110] Lawrence S.R., Shah K.M. (2024). Prospects and Current Challenges of Extracellular Vesicle-Based Biomarkers in Cancer. Biology.

[111] Batool S.M., Hsia T., Khanna S.K., Gamblin A.S., Rosenfeld Y., You D.G., Carter B.S., Balaj L. (2022). Decoding vesicle-based precision oncology in gliomas. Neuro-Oncol. Adv..

[112] Agnihotri T.G., Salave S., Shinde T., Srikanth I., Gyanani V., Haley J.C., Jain A. (2023). Understanding the role of endothelial cells in brain tumor formation and metastasis: A proposition to be explored for better therapy. J. Natl. Cancer Cent..

[113] Cathcart J., Pulkoski-Gross A., Cao J. (2015). Targeting Matrix Metalloproteinases in Cancer: Bringing New Life to Old Ideas. Genes Dis..

[114] Ganji M., Bakhshi S., Shoari A., Cohan R.A. (2023). Discovery of potential FGFR3 inhibitors via QSAR, pharmacophore modeling, virtual screening and molecular docking studies against bladder cancer. J. Transl. Med..

[115] Zhao Y., He X., Wan Q. (2024). Combined machine learning models, docking analysis, ADMET studies and molecular dynamics simulations for the design of novel FAK inhibitors against glioblastoma. BMC Chem..

[116] Fields G.B. (2019). The Rebirth of Matrix Metalloproteinase Inhibitors: Moving Beyond the Dogma. Cells.

[117] Hashimoto H., Takeuchi T., Komatsu K., Miyazaki K., Sato M., Higashi S. (2011). Structural basis for matrix metalloproteinase-2 (MMP-2)-selective inhibitory action of beta-amyloid precursor protein-derived inhibitor. J. Biol. Chem..

[118] Shoari A., Rasaee M.J., Kanavi M.R., Daraei B. (2019). Functional mimetic peptide discovery isolated by phage display interacts selectively to fibronectin domain and inhibits gelatinase. J. Cell Biochem..

[119] Lee H., Youn I., Demissie R., Vaid T.M., Che C.T., Azar D.T., Han K.Y. (2023). Identification of small molecule inhibitors against MMP-14 via High-Throughput screening. Bioorg. Med. Chem..

[120] Uhm J.H., Dooley N.P., Villemure J.G., Yong V.W. (1996). Glioma invasion in vitro: Regulation by matrix metalloprotease-2 and protein kinase C. Clin. Exp. Metastasis.

[121] Li X.Y., Huang G.H., Liu Q.K., Yang X.T., Wang K., Luo W.Z., Liang T.S., Yuan S.P., Zhen Y.W., Yan D.M. (2020). Porf-2 Inhibits Tumor Cell Migration Through the MMP-2/9 Signaling Pathway in Neuroblastoma and Glioma. Front. Oncol..

[122] Ye Y., Kuang X., Xie Z., Liang L., Zhang Z., Zhang Y., Ma F., Gao Q., Chang R., Lee H.H. (2020). Small-molecule MMP2/MMP9 inhibitor SB-3CT modulates tumor immune surveillance by regulating PD-L1. Genome Med..

[123] Hadass O., Tomlinson B.N., Gooyit M., Chen S.Y., Purdy J.J., Walker J.M., Zhang C.Y., Giritharan A.B., Purnell W., Robinson C.R. (2013). Selective Inhibition of Matrix Metalloproteinase-9 Attenuates Secondary Damage Resulting from Severe Traumatic Brain Injury. PLoS ONE.

[124] Thanh H.D., Lee S., Nguyen T.T., Huu T.N., Ahn E.J., Cho S.H., Kim M.S., Moon K.S., Jung C. (2024). Temozolomide promotes matrix metalloproteinase 9 expression through p38 MAPK and JNK pathways in glioblastoma cells. Sci. Rep..

[125] Zhong J., Shan W., Zuo Z. (2021). Norepinephrine inhibits migration and invasion of human glioblastoma cell cultures possibly via MMP-11 inhibition. Brain Res..

[126] Guan N., Huo X., Zhang Z., Zhang S., Luo J., Guo W. (2015). Ginsenoside Rh2 inhibits metastasis of glioblastoma multiforme through Akt-regulated MMP13. Tumour Biol..

[127] Takino T., Nakada M., Li Z., Yoshimoto T., Domoto T., Sato H. (2016). Tip60 regulates MT1-MMP transcription and invasion of glioblastoma cells through NF-kappaB pathway. Clin. Exp. Metastasis.

[128] Laronha H., Carpinteiro I., Portugal J., Azul A., Polido M., Petrova K.T., Salema-Oom M., Caldeira J. (2020). Challenges in Matrix Metalloproteinases Inhibition. Biomolecules.

[129] Taheri E., Raeeszadeh-Sarmazdeh M. (2025). Effect of TIMPs and their minimally engineered variants in blocking invasion and migration of brain cancer cells. Oncotarget.

[130] Kalantar M., Kalanther I., Kumar S., Buxton E.K., Raeeszadeh-Sarmazdeh M. (2024). Determining key residues of engineered scFv antibody variants with improved MMP-9 binding using deep sequencing and machine learning. Comput. Struct. Biotechnol. J..

[131] Kalantar M., Hilpert G.A., Mosca E.R., Raeeszadeh-Sarmazdeh M. (2024). Engineering metalloproteinase inhibitors: Tissue inhibitors of metalloproteinases or antibodies, that is the question. Curr. Opin. Biotechnol..

[132] Sullivan H.L., Liang Y., Worthington K., Luo C., Gianneschi N.C., Christman K.L. (2023). Enzyme-Responsive Nanoparticles for the Targeted Delivery of an MMP Inhibitor to Acute Myocardial Infarction. Biomacromolecules.

[133] Pourmasoumi P., Banihashemian S.A., Zamani F., Rasouli-Nia A., Mehrabani D., Karimi-Busheri F. (2024). Nanoparticle-Based Approaches in the Diagnosis and Treatment of Brain Tumors. J. Clin. Med..

[134] Parakh S., Nicolazzo J., Scott A.M., Gan H.K. (2021). Antibody Drug Conjugates in Glioblastoma—Is There a Future for Them?. Front. Oncol..

[135] Tanabe H., Suzuki T., Ohishi T., Isemura M., Nakamura Y., Unno K. (2023). Effects of Epigallocatechin-3-Gallate on Matrix Metalloproteinases in Terms of Its Anticancer Activity. Molecules.

[136] Kciuk M., Alam M., Ali N., Rashid S., Glowacka P., Sundaraj R., Celik I., Yahya E.B., Dubey A., Zerroug E. (2023). Epigallocatechin-3-Gallate Therapeutic Potential in Cancer: Mechanism of Action and Clinical Implications. Molecules.

[137] Asuthkar S., Velpula K.K., Chetty C., Gorantla B., Rao J.S. (2012). Epigenetic Regulation of miRNA-211 by MMP-9 Governs Glioma Cell Apoptosis, Chemosensitivity and Radiosensitivity. Oncotarget.

[138] Veeravalli K.K., Rao J.S. (2012). MMP-9 and uPAR regulated glioma cell migration. Cell Adhes. Migr..

[139] Zhao H., Xing F., Yuan J., Li Z., Zhang W. (2020). Sevoflurane inhibits migration and invasion of glioma cells via regulating miR-34a-5p/MMP-2 axis. Life Sci..

[140] Thakur V., Thakur V.S., Aguila B., Slepak T.I., Wang M., Song W., Konai M., Mobashery S., Chang M., Rana A.B. (2022). Targeting extracellular matrix remodeling sensitizes glioblastoma to ionizing radiation. Neuro-Oncol. Adv..

[141] Banerjee K., Núñez F.J., Haase S., McClellan B.L., Faisal S.M., Carney S.V., Yu J., Alghamri M.S., Asad A.S., Candia A.J.N. (2021). Current Approaches for Glioma Gene Therapy and Virotherapy. Front. Mol. Neurosci..

[142] Lara-Velazquez M., Alkharboosh R., Norton E.S., Ramirez-Loera C., Freeman W.D., Guerrero-Cazares H., Forte A.J., Quiñones-Hinojosa A., Sarabia-Estrada R. (2020). Chitosan-Based Non-viral Gene and Drug Delivery Systems for Brain Cancer. Front. Neurol..

[143] Chao C.N., Yang Y.H., Wu M.S., Chou M.C., Fang C.Y., Lin M.C., Tai C.K., Shen C.H., Chen P.L., Chang D.C. (2018). Gene therapy for human glioblastoma using neurotropic JC virus-like particles as a gene delivery vector. Sci. Rep..

[144] Hersh A.M., Alomari S., Tyler B.M. (2022). Crossing the Blood-Brain Barrier: Advances in Nanoparticle Technology for Drug Delivery in Neuro-Oncology. Int. J. Mol. Sci..

[145] Lim S.H., Yee G.T., Khang D. (2024). Nanoparticle-Based Combinational Strategies for Overcoming the Blood-Brain Barrier and Blood-Tumor Barrier. Int. J. Nanomed..

[146] Justo B.L., Jasiulionis M.G. (2021). Characteristics of TIMP1, CD63, and beta1-Integrin and the Functional Impact of Their Interaction in Cancer. Int. J. Mol. Sci..

[147] Su C.W., Lin C.W., Yang W.E., Yang S.F. (2019). TIMP-3 as a therapeutic target for cancer. Ther. Adv. Med. Oncol..

[148] Hernandez-Barrantes S., Shimura Y., Soloway P.D., Sang Q.A., Fridman R. (2001). Differential roles of TIMP-4 and TIMP-2 in pro-MMP-2 activation by MT1-MMP. Biochem. Biophys. Res. Commun..

[149] Han J., Jing Y., Han F., Sun P. (2021). Comprehensive analysis of expression, prognosis and immune infiltration for TIMPs in glioblastoma. BMC Neurol..

[150] Vandenbroucke R.E., Libert C. (2014). Is there new hope for therapeutic matrix metalloproteinase inhibition?. Nat. Rev. Drug Discov..

[151] Groft L.L., Muzik H., Rewcastle N.B., Johnston R.N., Knäuper V., Lafleur M.A., Forsyth P.A., Edwards D.R. (2001). Differential expression and localization of TIMP-1 and TIMP-4 in human gliomas. Br. J. Cancer.

[152] Waller V., Pruschy M. (2021). Combined Radiochemotherapy: Metalloproteinases Revisited. Front. Oncol..

[153] Winer A., Adams S., Mignatti P. (2018). Matrix Metalloproteinase Inhibitors in Cancer Therapy: Turning Past Failures Into Future Successes. Mol. Cancer Ther..

[154] Fields G.B. (2019). Mechanisms of Action of Novel Drugs Targeting Angiogenesis-Promoting Matrix Metalloproteinases. Front. Immunol..

[155] Melssen M.M., Sheybani N.D., Leick K.M., Slingluff C. (2023). Barriers to immune cell infiltration in tumors. J. Immunother. Cancer.

[156] Augoff K., Hryniewicz-Jankowska A., Tabola R., Stach K. (2022). MMP9: A Tough Target for Targeted Therapy for Cancer. Cancers.

[157] Ulasov I., Thaci B., Sarvaiya P., Yi R., Guo D., Auffinger B., Pytel P., Zhang L., Kim C.K., Borovjagin A. (2013). Inhibition of MMP14 potentiates the therapeutic effect of temozolomide and radiation in gliomas. Cancer Med..

[158] Gusev Y., Bhuvaneshwar K., Song L., Zenklusen J.C., Fine H., Madhavan S. (2018). The REMBRANDT study, a large collection of genomic data from brain cancer patients. Sci. Data.

[159] Duan H., Ren J.L., Wei S.Y., Yang Z.Y., Li C., Wang Z.N., Li M.C., Wei Z., Liu Y., Wang X.Q. (2024). Integrated analyses of multi-omic data derived from paired primary lung cancer and brain metastasis reveal the metabolic vulnerability as a novel therapeutic target. Genome Med..

[160] Shi Y.X., Zhang Q.L., Mei J., Liu J.H. (2023). Editorial: Multi-omics analysis in tumor microenvironment and tumor heterogeneity. Front. Genet..

[161] Wang Y.C., Chen R., Wa Y., Ding S.K., Yang Y.J., Liao J.B., Tong L., Xiao G.L. (2022). Tumor Immune Microenvironment and Immunotherapy in Brain Metastasis from Non-Small Cell Lung Cancer. Front. Immunol..

[162] Czajka-Francuz P., Prendes M.J., Mankan A., Quintana A., Pabla S., Ramkissoon S., Jensen T.J., Peiró S., Severson E.A., Achyut B.R. (2023). Mechanisms of immune modulation in the tumor microenvironment and implications for targeted therapy. Front. Oncol..

[163] Tang L., Feng Y.C., Gao S., Mu Q.C., Liu C.Y. (2021). Nanotherapeutics Overcoming the Blood-Brain Barrier for Glioblastoma Treatment. Front. Pharmacol..

[164] Wang Q.Y., Cui H.J., Gan N., Ma X.H., Ren W.Z., Wu A.G. (2022). Recent advances in matrix metalloproteinases-responsive nanoprobes for cancer diagnosis and therapy. Rev. Anal. Chem..

[165] Chistiakov D.A., Chekhonin V.P. (2014). Extracellular vesicles shed by glioma cells: Pathogenic role and clinical value. Tumor Biol..

[166] Raeeszadeh-Sarmazdeh M., Coban M., Mahajan S., Hockla A., Sankaran B., Downey G.P., Radisky D.C., Radisky E.S. (2022). Engineering of tissue inhibitor of metalloproteinases TIMP-1 for fine discrimination between closely related stromelysins MMP-3 and MMP-10. J. Biol. Chem..

[167] Ashja Ardalan A., Khalili-Tanha G., Shoari A. (2024). Shaping the Landscape of Lung Cancer: The Role and Therapeutic Potential of Matrix Metalloproteinases. Int. J. Transl. Med..

